# Experimental Investigation of Water Vapor Concentration on Fracture Properties of Asphalt Concrete

**DOI:** 10.3390/ma17133289

**Published:** 2024-07-03

**Authors:** Yu Chen, Tingting Huang, Xuqing Wen, Kai Zhang, Zhengang Li

**Affiliations:** 1 School of Transportation and Logistics Engineering, Wuhan University of Technology, Hubei Highway Engineering Research Center, Wuhan 430063, China; yu.chen@whut.edu.cn (Y.C.); huangtingting@whut.edu.cn (T.H.);; 2 Jiangxi Provincial Communications Investment Group Co., Ltd. Project Construction Management Company, Nanchang 330200, China; 3 Jiangxi Transportation Institute Co., Ltd., 809 Jinsha Avenue, Nanchang 330200, China; 4 Wuhan Dongxihu District Transportation Bureau, Wuhan 430040, China

**Keywords:** asphalt concrete, water vapor concentration, relative humidity, fracture energy, tensile strength, semi-circular bending (SCB) test

## Abstract

The effect of moisture on the fracture resistance of asphalt concrete is a significant concern in pavement engineering. To investigate the effect of the water vapor concentration on the fracture properties of asphalt concrete, this study first designed a humidity conditioning program at the relative humidity (*RH*) levels of 2%, 50%, 80%, and 100% for the three types of asphalt concrete mixtures (AC-13C, AC-20C, and AC-25C).The finite element model was developed to simulate the water vapor diffusion and determine the duration of the conditioning period. The semi-circular bending (SCB) test was then performed at varying temperatures of 5 °C, 15 °C, and 25 °C to evaluate the fracture energy and tensile strength of the humidity-conditioned specimens. The test results showed that the increasing temperature and the *RH* levels resulted in a lower peak load but greater displacement of the mixtures. Both the fracture energy and tensile strength tended to diminish with the rising temperature. It was also found that moisture had a significant effect on the tensile strength and fracture energy of asphalt concrete. Specifically, as the *RH* level increased from 2% to 100% (i.e., the water vapor concentration rose from 0.35 g/m^3^ to 17.27 g/m^3^), the tensile strength of the three types of mixtures was reduced by 34.84% on average, which revealed that the water vapor led to the loss of adhesion and cohesion within the mixture. The genetic expression programming (GEP) model was developed to quantify the effect of water vapor concentrations and temperature on the fracture indices.

## 1. Introduction

Moisture has a crucial impact on the performance and durability of asphalt concrete. Moisture infiltration into the asphalt concrete mixture can weaken the adhesion between aggregates and bitumen and lead to stripping, which is often referred to as moisture damage [1]. Forms of water in the pavement structure mainly exist as liquid and water vapor. Existing studies have found that water vapor is easier to diffuse or accumulate in the air void of asphalt concrete compared to liquid [2,3]. In this way, water vapor is regarded as the primary moisture form that affects the performance of asphalt concrete. Since water vapor infiltration can cause the separation of the bitumen and aggregates, it also has an effect on crack initiation and propagation in asphalt concrete. Therefore, it is imperative to study the effect of water vapor on the fracture resistance of asphalt concrete.

To study the influence of water vapor, humidity conditioning is required to ensure that the asphalt concrete mixture is conditioned at varying water vapor concentrations. Existing studies have mainly focused on the moisture susceptibility of the asphalt concrete mixture using methods such as the boiling test [4], freeze–dry cycles [5,6], the vacuum saturation method [7], and so forth [8]. Compared to moisture conditioning, humidity conditioning, which controls the water vapor concentration and humidity level, is more complex and requires more sophisticated devices.

Some existing studies have attempted different methods to achieve controlled relative humidity (*RH*) levels. Hossain and Tarefder [9] utilized an aqueous solution of potassium acetate (CH_3_COOK), potassium carbonate (K_2_CO_3_), and sodium chloride (NaCl) in enclosed desiccators to create a constant *RH* level of 25%, 49%, and 71%, respectively, for shear pull-off test specimens by following the ASTM E104 standard [10]. Tong et al. utilized a desiccant and distilled water in a vacuum desiccator to achieve the *RH* level of 0% and 100%, respectively [11]. The controlled *RH* level by a saturated salt solution requires sophisticated devices to maintain sealability and stable ambient temperature. Cheng et al. utilized a universal sorption device to measure the amount of water vapor absorbed in the mastic [12] while Xi et al. employed an environmental chamber to maintain a steady water vapor environment for humidity conditioning [13]. 

To study the effect of moisture on asphalt concrete, previous studies commonly conducted performance tests and compared the performance parameters of the asphalt mixture before and after moisture conditioning. Lytton et al. conducted a dynamic mechanical analysis (DMA) test to evaluate the rate of damage accumulation and found that moisture damage resistance was related to bond energy calculation [14]. Lee et al. performed a dynamic modulus test to evaluate the moisture susceptibility of the asphalt mixture [15] while Wang et al. performed a uniaxial penetration strength test to assess moisture sensitivity based on dynamic water environmental conditioning [16]. Akentuna et al. evaluated the effectiveness of different laboratory mechanical tests such as the loaded wheel tracking test, modified Lottman, and semi-circular bending test to study the moisture sensitivity of asphalt mixtures [17].

Fracture resistance is one of the most significant properties of asphalt concrete. The evaluation of its crack resistance relies on various fracture indices, which are divided into mechanical parameters and energy index (e.g., fracture energy), based on the different laboratory tests. Previous studies primarily focused on the effect of temperature, loading mode (e.g., loading rate), specimen geometry, and mixture composition on the fracture resistance of asphalt mixtures [18,19]. For instance, Li et al. conducted the semi-circular bending (SCB) test to evaluate the fracture resistance of the six types of asphalt mixtures with different binder types, binder modifiers, aggregate type, air voids, loading rate, initial notch length, and low-temperature level [20].

Some researchers have increasingly focused on the effect of moisture on the fracture resistance of the asphalt mixture. Specifically, the effect of moisture on the fracture resistance of asphalt concrete was investigated using moisture conditioning methods such as water baths, freeze–thaw cycles, and saline solutions. Karimi et al. performed moisture conditioning in a water bath and moisture damage at different levels on the specimens and conducted the SCB test to evaluate the effect of moisture and freeze–thaw damage on the fracture properties [21]. Fakhri and Ahmadi considered two aging levels and the freeze–thaw cycles on the asphalt mixture fabricated with reclaimed asphalt pavement (RAP) and coarse steel slag aggregate (SSA) and conducted the SCB test to investigate the fracture resistance [22]. Zhang et al. utilized chloride solutions to perform dry–wet cycles on the asphalt mixture and then conducted the splitting test to study the impact of different salty and moisture conditions on the mechanical properties of the mixtures [23]. 

However, there are limited studies considering humidity conditioning to control the water vapor concentration and its effect on the fracture resistance of asphalt concrete. Therefore, there is a need to explore the impact of humidity conditioning on asphalt concrete mixtures, as the water vapor diffuses and accumulates more easily in mixtures compared to liquid water, resulting in different severities of moisture damage. 

Therefore, this study aimed to investigate the effect of water vapor concentration on the fracture properties of the asphalt concrete mixtures. The asphalt concrete specimens were humidity-conditioned at various *RH* levels of 2%, 50%, 80%, and 100% in the form of water vapor, and the SCB test was then conducted to assess the fracture properties of the humidity-conditioned specimens. 

## 2. Materials and Methods

### 2.1. Materials

The asphalt binders used in this study were styrene-butadiene-styrene (SBS)-modified asphalt and neat 70# binder. To ensure that the asphalt concrete mixture meets the standard specification, binder tests were conducted in accordance with the Chinese specification JTG E20-2011 [24] to obtain the properties (or technical indices) of the two types of binder such as penetration, ductility, softening point, and density. Moreover, the thin film oven test (TFOT) was conducted to evaluate the durability and aging characteristics of the binder by simulating the short-term aging of the binder. These binder test results met the technical index requirements in Chinese specification JTG F40-2004 [25]. Table 1 and Table 2 present the binder test results for the SBS-modified binder and neat 70# binder, respectively.

### 2.2. Sample Preparation

**Figure 1:**
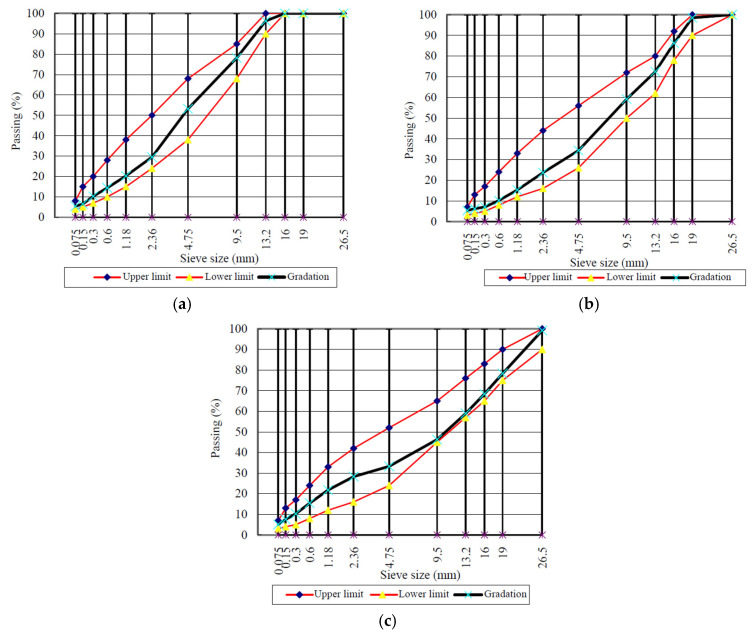
Aggregate gradation for (**a**) AC-13; (**b**) AC-20C; (**c**) AC-25C mixture.

The test sample was prepared for humidity conditioning and then the semi-circular bending (SCB) test. Two typical types of aggregates including limestone and diabase were used for the preparation of the test sample. Three dense-graded asphalt concrete mixtures of AC-13C, AC-20C, and AC-25C were considered in this study based on the sponsored project. The AC-13C mixture used the SBS-modified asphalt and diabase, the AC-20C mixture used the SBS-modified asphalt and limestone, and the AC-25C mixture used the neat 70# asphalt and limestone. The aggregate gradation of the three types of asphalt concrete mixture is shown in Figure 1. The Marshall mix design of the three types of asphalt concrete mixture was conducted by following Chinese specification JTG E20-2011 [24] to determine the optimal asphalt content. The standard Marshall specimens based on the optimized aggregate gradation as shown in Figure 1 were fabricated for the Marshall stability test and volumetric mix design. The test results of the Marshall stability test and volumetric properties of the asphalt concrete mixtures met the requirements in Chinese specification JTG F40-2004 [25], as presented in Table 3. The optimal asphalt contents of the three asphalt concrete mixtures were obtained as 4.8%, 4.5, and 3.9%, respectively, as shown in Table 3. 

**Figure 2:**
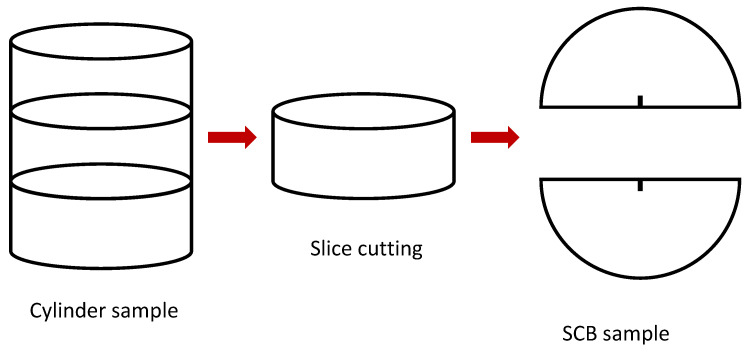
Sample preparation procedure for the SCB test.

Based on the aggregate gradation and optimal asphalt content, the test specimens were compacted with a Superpave Gyratory Compactor (SGC) to obtain a cylinder sample with a diameter of 150 mm and a height of 180 mm. For the center of the cylinder samples, cylinder slices with a diameter of 150 mm and a thickness of 50 mm were obtained and then cut into two identical “halves” as the SCB test specimens. The specimens had a notch cut in the middle as the pre-existing cracking. The notch length and width were selected as 10 mm and 1 mm, respectively. Specifically, the SCB specimen with and without a notch was fabricated to obtain the fracture energy and tensile strength, respectively [26]. The preparation procedure of the specimen for the SCB test is depicted in Figure 2. In order to reduce the measurement error, three parallel samples were fabricated for each test condition of temperature, water vapor concentration, and mixture type, etc. In total, we prepared 216 SCB test specimens to ensure the reliability of our test results. 

### 2.3. Humidity Conditioning Program

In order to investigate the effect of water vapor concentration on the fracture behavior of asphalt concrete, the humidity conditioning program for the SCB test specimen was performed. Relative humidity (*RH*) is most widely used to represent humidity conditioning and refers to the percentage value of water pressure in the air and the saturated water pressure at the same temperature. The *RH* and water vapor concentration can be converted from the ideal gas law formula:

(1) $$RH=\frac{RT}{P_{T}m_{H_{2}O}}\cdot C$$

where RH is the relative humidity, %; R is the general gas constant, 8.314 J/mol·K; T is the Kelvin temperature, K; $m_{H_{2}O}$ is the molar mass of water, 18.015 g/mol; $P_{T}$ is the saturated water vapor pressure, 2336.33 Pa at 20 °C; C is the water vapor concentration, g/m^3^.

**Figure 3:**
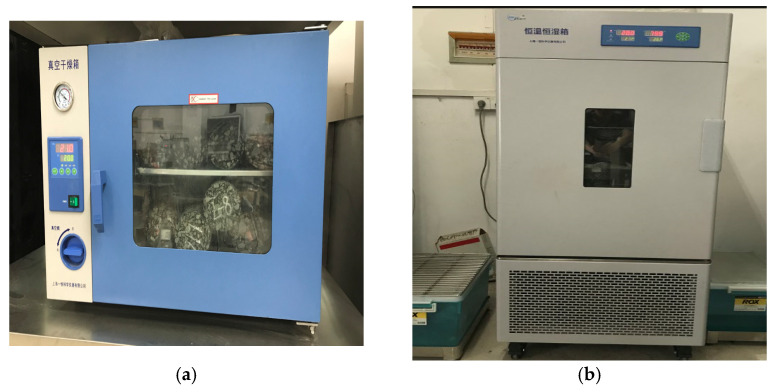
Humidity conditioning apparatus for different *RH* levels: (**a**) vacuum drying oven; (**b**) constant temperature and humidity chamber.

To cover a wide range of realistic environmental conditions, this study selected the *RH* levels of 2%, 50%, 80%, and 100% at a temperature of 20 °C as the humidity curing condition. At the *RH* level of 2%, asphalt concrete is subjected to the dry condition, while at the *RH* level of 100%, it is subjected to the saturation condition. A humidity chamber and vacuum drying oven were used for humidity conditioning under different *RH* levels, as shown in Figure 3. To reach the *RH* level of 2%, the negative pressure environment in the humidity chamber was made by pumping out air, and the dry desiccant of phosphorus pentoxide (P_2_O_5_) power was placed into the open glass container to absorb the water in the humidity chamber. Since the dry desiccant cannot fully absorb the water vapor, the *RH* level was maintained at an *RH* level of 2%, which is why the level was selected as the dry condition. 

**Figure 4:**
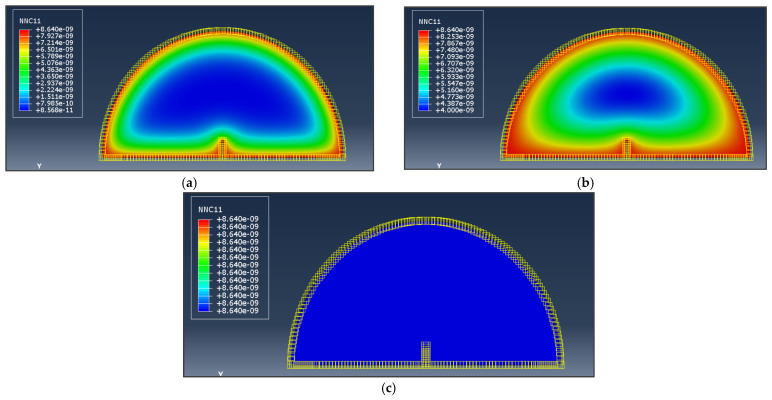
Water vapor diffusion in the simulation for (**a**) early stage; (**b**) middle stage; (**c**) final stage.

The humidity conditioning program designed in this study requires both the *RH* levels and the humidity curing period, which refers to the duration of time during which asphalt concrete specimens are conditioned at the specific *RH* level to reach moisture equilibrium within the specimens. To determine the humidity curing period, the finite element model was built to simulate the water vapor distribution in asphalt concrete and determine the period required to achieve the specified water vapor concentration. The initial water vapor distribution and boundary condition were defined, and the water vapor diffusion was simulated, as referenced in [27]. Figure 4 shows the three stages of water vapor diffusion. The difference in water vapor concentration between the boundary and interior of the virtual specimen led to the continuous diffusion of water vapor from the boundary to the center until the equilibrium was reached. Based on the simulation results and to avoid influencing factors such as aging, the humidity curing period of 4 months was chosen for the SCB test specimens. 

### 2.4. Semi-Circular Bending Test

**Figure 5:**
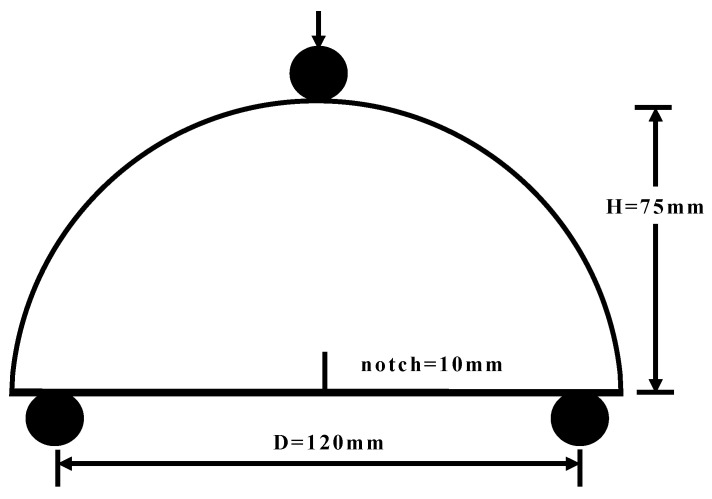
The SCB test configuration.

The SCB test was conducted to evaluate the fracture properties of the asphalt concrete mixtures under different humidity and temperature conditions. The SCB specimens were subjected to the temperatures of 5 °C, 15 °C, and 25 °C. The load was applied at the constant displacement rate of 2 mm/min. The SCB test setup and procedure followed the AASHTO TP105-13 protocol. The SCB test was set up as shown in Figure 5. The SCB test specimen was supported at two rollers and loaded at a single roller in the middle of the specimen. The length of the support roller for the SCB test was defined as the fraction of the test specimen diameter (D). A typical value of length is approximately 0.8D (i.e., 120 mm) [28]. 

To maintain the *RH* level during the SCB test, the dry agent of P_2_O_5_ power was placed into the environment chamber to absorb water vapor for the *RH* level of 2%. A humidifier was installed in the environment chamber to maintain the stable humidity levels for the *RH* levels of 50%, 80%, and 100%. 

In this study, the SCB test under different *RH* levels was conducted to evaluate the fracture resistance and tensile strength of asphalt concrete and investigate the water vapor concentration (or the *RH* level) on the fracture behavior of the asphalt mixture. The fracture energy is used to characterize the crack resistance of asphalt concrete. In the SCB test, the fracture energy indicates the energy required for the crack propagation. The area under the load–displacement curve is the fracture work for asphalt concrete completely fractured. The fracture energy is calculated as the ratio of the fracture work to the effective fracture area, as shown in Equation (2).

(2) $$G_{f}=\frac{W_{f}}{A_{act}}$$

where $G_{f}$ is the fracture energy, mJ/mm^2^; $W_{f}$ is the fracture work; $A_{act}\;$ is the effective fracture area, $A_{act}=\left(r-c\right)\times t$; r is the specimen radius, mm; c is the notch length, mm; t is the specimen thickness, mm.

Tensile strength is another crucial indicator of the crack resistance of asphalt concrete. The crack initiates and propagates when the tensile stress exceeds the tensile strength. According to the literature [29], the tensile strength can be obtained by the SCB test using the specimen without a notch. Tensile strength refers to the maximum tensile stress calculated at the center bottom of the SCB specimen by the following:

(3) $$\sigma_{t}=\frac{3PL}{2th^{2}}=\frac{6PL}{tD^{2}}$$

where $\sigma_{t}$ is the tensile strength, MPa; P is the peak load, N; L is the length of support roller, mm; h is the height of the specimen, mm; t is the specimen thickness, mm; D is the specimen diameter, mm.

## 3. Results and Discussion

### 3.1. Load–Displacement Curve

**Figure 6:**
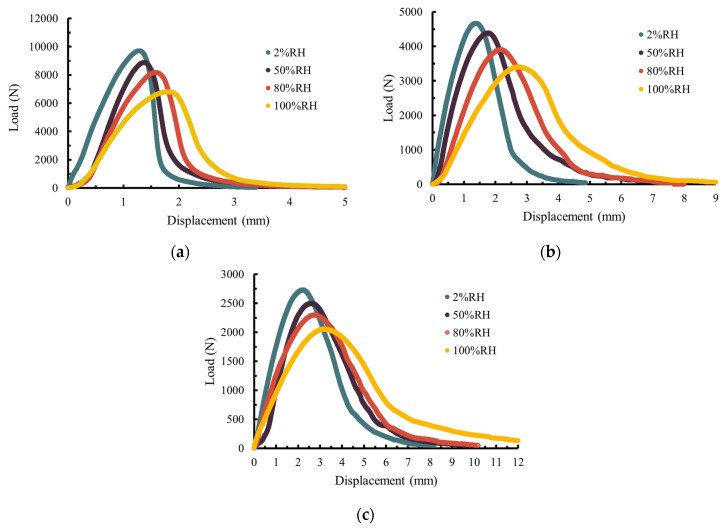
Load–displacement curve at different test temperatures for AC-13C at temperature of (**a**) 5 °C; (**b**) 15 °C; (**c**) 25 °C.

Figure 6 shows the load–displacement curve under different *RH* and temperature levels for AC-13C. It was found that the curve showed the viscoelastic behavior of the specimen at an intermediate temperature (5 °C~15 °C). With the increase in loading time at the constant displacement rate, the load on the SCB specimen gradually increased and the notched crack continued to propagate with the accumulation of the fracture work. When the load reached the peak value, the SCB specimen became unstable, which led the notched crack to rapidly propagate upward. The load decreased significantly after the peak, which indicates that the SCB specimen had fractured and could not sustain the substantial loading. 

Figure 6 also shows that as the temperature increased, the peak load of the asphalt concrete mixture decreased, while the maximum displacement increased correspondingly, which indicates that the high temperature can reduce the bearing capacity of the asphalt concrete mixture. At the temperature of 5 °C, the load–displacement curve after the peak load dropped steeply, which indicates a decrease in the viscous deformation of the specimen. At the temperatures of 15 °C and 15 °C, the curve after the peak load dropped gradually and showed good toughness. 

Moreover, Figure 6 shows that as the water vapor concentration (or the *RH* level) increased, the peak load of the asphalt concrete mixture showed a downward trend, which indicates that the high water vapor concentration can lead to a decrease in the bearing capacity of the mixture. The maximum displacement increased correspondingly with the rising temperature, which indicates that under a sufficient humidity curing period, the specimen becomes softer and more ductile. 

### 3.2. Fracture Energy

**Figure 7:**
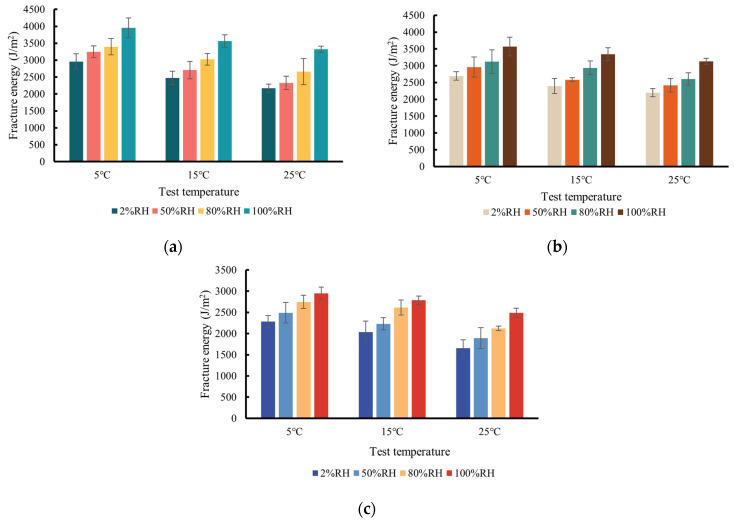
Fracture energy vs. test temperature at different *RH* levels for (**a**) AC-13; (**b**) AC-20C; (**c**) AC-25C mixture.

At the intermediate temperature, the mixture behavior is governed by the viscoelastic fracture mechanics. Fracture energy can be used to characterize the crack resistance of the mixture. Figure 7 shows that the fracture energy had a downward trend as the temperature increased for the three types of mixtures. The AC-13C mixture had the highest fracture energy and showed the best toughness due to it using the SBS-modified asphalt and had a higher asphalt content to withstand larger deformation. 

It was observed from Figure 7 that as the *RH* level (or the water vapor concentration) increased, the fracture energy of the three types of mixtures increased, which seems counter-intuitive. This is because, as noted in Figure 6, that the elevated *RH* level increased the ductility of the asphalt concrete mixture, which possibly led to a larger fracture energy. Another reason is that the water vapor may alter the internal pore pressure of the mixture and help to distribute the stress during loading. 

### 3.3. Tensile Strength

**Figure 8:**
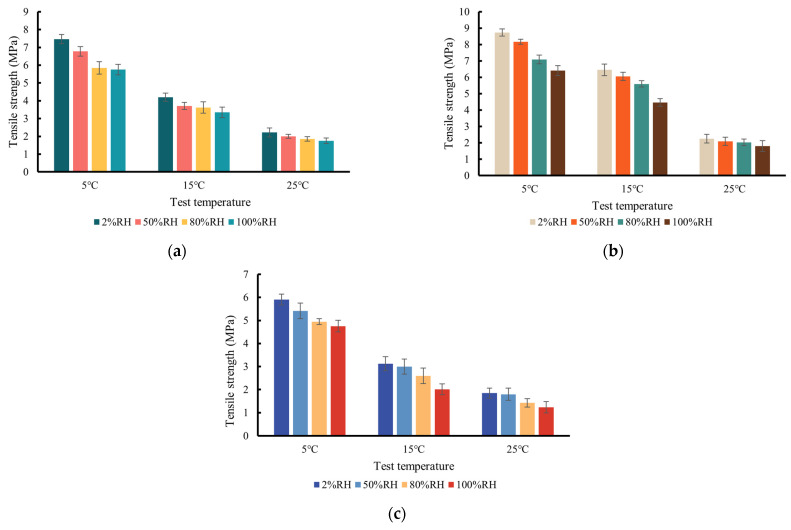
Tensile strength vs. test temperature at different *RH* levels for (**a**) AC-13; (**b**) AC-20C; (**c**) AC-25C mixture.

Tensile strength is one of the significant parameters in which to evaluate the fracture properties of the asphalt concrete mixture. Figure 8 shows the tensile strength obtained by Equation (3) at varying temperatures and *RH* levels (or water vapor concentration). It is known that the tensile strength is primarily influenced by the peak load and the specimen dimension. It was noted in Figure 8 that the tensile strength showed a downward trend, which is consistent with the peak load. As the temperature increased, the tensile strength of the three types of mixtures declined, which indicates that the adhesion of the aggregate–asphalt interface dropped with higher temperature. 

Figure 8 also shows that as the *RH* level rose from 2% to 100% (i.e., the water vapor concentration rose from 0.35 g/m^3^ to 17.27 g/m^3^), the tensile strength of the three types of mixtures was reduced by 34.84% on average. This shows that the water vapor resulted in the loss of adhesion and cohesion within the mixture. 

### 3.4. Regression Analysis

This study correlated the water vapor concentration and temperature with the fracture indices to quantify their effects on the fracture resistance of asphalt concrete. The fracture indices including the fracture energy and tensile strength were calculated from the SCB tests. The genetic expression programming (GEP) model was adopted to perform this correlation due to its advantages in regression analysis with small datasets. 

**Figure 9:**
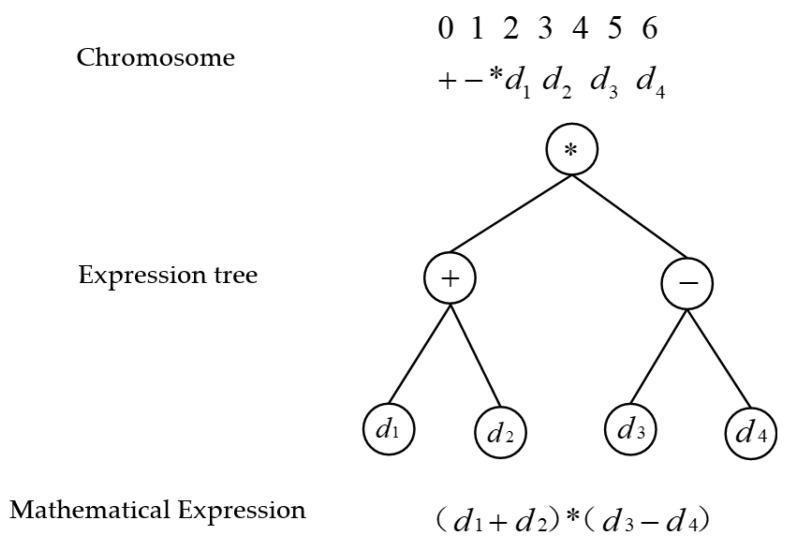
Schematic diagram of the GEP model structure.

The GEP is an evolutionary algorithm to develop models without pre-defined expression and can evolve the form of the expression itself along with its parameters [30]. The structure of the GEP model includes chromosome and expression trees. The chromosome is composed of genes coded as a fixed-length string such as “0123456+−*d_1_d_2_d_3_d_4_”. Chromosomes can be translated into expression trees and evolved by gene operators such as mutation (*), subtraction (−), and recombination (+), from one generation to the next. The mathematical expression is developed and optimized via evolution. The expression is composed of different variables (i.e., “d_1_”, “d_2_”, “d_3_”, and “d_4_”) and operators (i.e., “+”, “−”, and “*”). Figure 9 depicts the schematic diagram of the GEP model structures.

The GEP model was developed to quantify the effect of water vapor concentration and temperature on the fracture properties of asphalt concrete. The performance of the GEP model was evaluated by using the root relative square error (RRSE) and R-squared, as calculated below:

(4) $$RRSE=\sqrt{\frac{\sum_{i=1}^{n}\;(y_{i}-{\hat{y}}_{i}{)}^{2}}{\sum_{i=1}^{n}\;(y_{i}-\bar{y}{)}^{2}}}$$

(5) $$R^{2}=1-\frac{\sum_{i=1}^{n}{\left(y_{i}-{\hat{y}}_{i}\right)}^{2}}{\sum_{i=1}^{n}{\left(y_{i}-\bar{y}\right)}^{2}}$$

where $R^{2}$ is the coefficient of determination; $y_{i}$ is the *i*-th tested value; ${\hat{y}}_{i}\;$ is the *i*-th predicted value or fitted value; $\bar{y}\;$ is the mean value of tested values; n is the total number of tested values. Both the RRSE and R-squared value are model metrics used to evaluate model performance. The R-squared value commonly falls into the range between 0 and 1. The R-squared value close to 1 means more accurate model prediction performance. The lower the RRSE value, the better the model’s predictive power. 

Table 4 presents the mathematical expressions derived from the GEP model based on the SCB test results for the fracture energy and ($y_{1}$) tensile strength ($y_{2}$) where $x_{1}$ and $x_{2}$ refer to the temperature (°C) and water vapor concentration (g/m^3^), respectively. It can be seen from Table 4 that the R-squared values for the GEP models across the three types of mixtures exceeded 0.94, which means that the GEP models showed good agreement with the fracture energy and tensile strength obtained from the SCB test. Therefore, the GEP models are applicable for different mixture types (AC-13C, AC-20C, and AC-25C), but may not extend to other types of materials due to variations in binder type, aggregate characteristics, and mixture compositions.

## 4. Conclusions

In this study, the SCB test was performed to investigate the effect of the water vapor concentration (or the *RH* level) on the fracture properties of asphalt concrete. Three types of mixtures (AC-13C, AC-20C, and AC-25C) were utilized. The mixtures were humidity conditioned at varying *RH* levels of 2%, 50%, 80%, and 100% prior to the SCB test. The two significant parameters, that is, the fracture energy and tensile strength, were obtained to characterize the fracture properties of the mixtures. Based on the SCB test results, the main findings of this study can be drawn as follows:

- The humidity conditioning program was designed for the SCB specimens to study the impact of the *RH* levels of 2%, 50%, 80%, and 100% at 20 °C. The duration of the humidity curing period was determined by the finite element model, which simulated the water vapor diffusion until the equilibrium was reached.
- The load–displacement curve shows that the increase in *RH* level from 2% to 100% or the elevated temperature from 5 °C to 25 °C decreased the peak load but increased the displacement of the mixtures, which implies that the water vapor or higher temperature reduced the bearing capacity of mixture but improved the mixture toughness.
- The fracture energy showed a downward trend as the temperature increased for the three types of mixtures. Specifically, the AC-13C mixture had the highest fracture energy and showed the best toughness due to its use of the SBS-modified asphalt and higher asphalt content. Therefore, the use modified binders such as SBS-modified asphalt is recommended to enhance the durability of asphalt concrete, especially in regions with high humidity and temperature variations.
- With the rising *RH* levels, the fracture energy of the three types of mixtures increased since the water vapor enhanced the ductility of the mixture to some extent. Another reason is that the water vapor may alter the internal pore pressure of the mixture and help distribute the stress during loading.
- The tensile strength of the three types of mixtures declined with the increase in temperature, which indicates a reduction in the adhesion of the aggregate–asphalt interface with the higher temperature.
- As the *RH* level rose from 2% to 100% (i.e., the water vapor concentration rose from 0.35 g/m^3^ to 17.27 g/m^3^), the tensile strength of the three types of mixtures was reduced by 34.84% on average, which revealed that the water vapor led to the loss of adhesion and cohesion within the mixture.
- The GEP models were developed to quantify the effect of the water vapor concentration and temperature on the fracture indices. The R-squared values for the GEP models across the three types of mixtures were above 0.94, which indicates a good agreement between the GEP models and the fracture energy and tensile strength obtained from the SCB test.

The investigation in this study was restricted to the asphalt concrete mixtures with the SBS-modified binder and neat 70# binder. In future work, more binder types such as the modified binder with an anti-stripping additive will be utilized for humidity conditions and the SCB test. Moreover, the SCB tests will conducted on a wide variety of mixture types to enrich the data for the GEP model, thereby making it applicable to a broader range of mixtures. 

## Figures and Tables

**Table 1:** Test properties of the SBS-modified binder.

Properties		Unit	Test Value	Specification
Penetration (25 °C, 5 s, 100 g)		0.1 mm	55.0	40~60
Ductility (5 °C, 5 cm/min)		cm	27.0	≥20
Softening point (T_R&B_)		°C	76	≥75
Density		g/cm^3^	1.03	-
TFOT	Loss in mass	%	0.10	≤±1.0
	Reduction penetration (25 °C)	%	71.0	≥65
	Ductility (5 °C, 5 cm/min)	cm	17.0	≥15

**Table 2:** Test properties of the neat 70# binder.

Properties		Unit	Test Value	Specification
Penetration (25 °C, 5 s, 100 g)		0.1 mm	68.0	60~80
Ductility (5 °C, 5 cm/min)		cm	36.0	≥20
Softening point (T_R&B_)		°C	47.0	≥46
Density		g/cm^3^	1.04	-
TFOT residue	Loss in mass	%	0.03	≤±0.8
	Reduction penetration (25 °C)	%	75.0	≥61
	Ductility (10 °C, 5 cm/min)	cm	15.0	≥6

**Table 3:** Marshall stability test results.

Mixture Properties	AC-13C		AC-20C		AC-25C	
	Value	Specification	Value	Specification	Value	Specification
Optimal asphalt content	4.8	—	4.5	—	3.9	—
Bulk specific gravity (g/cm^3^)	2.412	—	2.457	—	2.492	—
Maximum specific gravity (g/cm^3^)	2.694	—	2.571	—	2.586	—
Air void (V_a_)/%	4.0	3~5	4.4	4~6	5.2	4~6
Air void in mineral aggregate (VMA)/%	14.6	≥14	14.0	≥13	12.1	≥12
Air void filled with asphalt (VFA)/%	71.3	65~75	68.4	65~75	69.7	65~75
Marshall Stability/kN	18.2	≥8	14.7	≥8	11.5	≥8
Flow value/0.1 mm	29.54	15~50	38.4	15~50	25.5	15~50

**Table 4:** GEP model results for fracture indices.

Mixture Type	Fracture Index	Expression	$R^{2}$	RRSE
AC-13C	Fracture energy	*y*_1_ = 0.88*x*_1_^2^ − 67.19*x*_1_ + 4.65*x*_2_^2^ − 15.81*x*_2_ + 3204.96	0.9821	0.0488
	Tensile strength	*y*_2_ = 0.005*x*_1_^2^ − 0.371*x*_1_ − 0.058*x*_2_ + 8.799	0.9431	0.0317
AC-20C	Fracture energy	*y*_1_ = −0.54*x*_1_^2^ − 16.85*x*_1_ + 3.93*x*_2_^2^ − 2.53*x*_2_ + 2592.53	0.9842	0.0465
	Tensile strength	*y*_2_ = −0.008*x*_1_^2^ − 0.032*x*_1_ − 0.006*x*_2_^2^ + 0.015*x*_2_ + 8.672	0.9560	0.0414
AC-25C	Fracture energy	*y*_1_ = −1.64*x*_1_^2^ + 17.13*x*_1_ + 2.44*x*_2_^2^ + 7.57*x*_2_ + 2117.36	0.9798	0.0394
	Tensile strength	*y*_2_ = 0.007*x*_1_^2^ − 0.404*x*_1_ − 0.003*x*_2_^2^ + 0.001*x*_2_ + 7.545	0.9491	0.0271

## Data Availability

All data, models, and codes generated or used in this study are included in the submitted manuscript.
